# Addressing hidden tensions and grey areas of general practice: a qualitative study of the experiences of newly qualified GPs attending a course on generalist medicine

**DOI:** 10.3399/BJGP.2023.0514

**Published:** 2024-08-20

**Authors:** Myriam Dell’Olio, Joanne Reeve

**Affiliations:** Academy of Primary Care, Hull York Medical School, Hull, UK.; Academy of Primary Care, Hull York Medical School, Hull, UK.

**Keywords:** continuing professional development, general practice, health workforce, practice organisation, primary health care, qualitative research

## Abstract

**Background:**

Generalist approaches can help address several challenges facing today’s primary care. However, GPs report insufficient support to deliver advanced generalist medicine (AGM) in daily practice, struggling within a healthcare system that imposes strict adherence to single-disease focused guidelines.

**Aim:**

To examine the professional and educational experiences of newly qualified GPs attending a course on AGM to understand how to redesign primary care systems to support their generalist work.

**Design and setting:**

This was a qualitative study focusing on AGM in UK general practice (England), conducted in the context of the research evaluation of an online career development programme on AGM.

**Method:**

We conducted 36 interviews and six focus groups with newly qualified GPs attending an online career development programme on AGM, and analysed data using framework analysis.

**Results:**

Three tensions experienced by the participants were identified: tension between realistic and idealistic practice; tension between different decision-making paradigms; and tension in the formation of the GPs’ professional identities. These were owing to grey areas of practice deeply rooted in primary care systems — namely areas of work not adequately addressed by current education and service design.

**Conclusion:**

Our findings have implications for tackling the general practice workforce crisis, highlighting that solutions targeting individual problems will not suffice by themselves. By making visible the grey areas of everyday general practice, we describe the changes needed to target tensions as described by the GPs in this study to ultimately enable, enhance and make visible the complex work of generalist medicine.

## Introduction

Healthcare systems are grappling with complex issues such as chronic conditions and multimorbidity, long COVID, and wider challenges posed by climate change, the energy crisis, socioeconomic inequalities, and poverty.^1^^–^4 Whereas it has been argued that the NHS needs more generalist medicine to address current healthcare challenges,^5^ we continue to misunderstand what the generalist role is.^6^ Advanced medical generalism is not simply the coordinated delivery of many different types of specialist medicine.^7^ It is a distinct form of medical practice able to critically generate, use, and appraise a whole-person**s** understanding of illness in context.^8^ It relies not simply on clinicians who know a little bit about lots of different diseases but on having practitioners trained and skilled to critically and creatively adapt and apply what they know to address a complex problem in context.^9^ The Royal College of General Practitioners defined GPs as consultants in general practice medicine, with expertise in whole-person medical care and managing complexity, uncertainty, and risk.^10^ But this definition does not explain how GP consultants fulfil that role.

Our misunderstanding around the generalist role brings up practical challenges to its implementation, as we are not providing our future workforce with the tools and skills they need to manage the complexity of modern healthcare practice.^11^ Part of the issue lies in how we understand the work to be done, and by implication how we define best practice. In today’s healthcare, this is defined by the principles of evidence-based medicine (EBM). EBM is defined as: *‘the conscientious, explicit, and judicious use of current best evidence in making decisions about the care of individual patients’*^12^ and is the paradigm that shapes understanding of best practice in the healthcare context. A paradigm is a set of theories, laws, and applications that define model problems and solutions for a community of practitioners.^13^ For instance, EBM privileges evidence derived from scientific studies to inform the decisions made by health professionals and their patients. Whereas we are seeing a rise in more interpretive paradigms of healthcare practice, which focus on the critical examination of patient narratives to guide clinical decision making,^14^^–^16 EBM’s emphasis on external scientific data is constraining clinicians’ access to patients’ narratives and values,^17^ hence, limiting their capacity to deliver generalist care. Primary care is becoming unsustainable, failing to properly address the complex challenges that doctors and patients are facing.^18^^,^^19^

**Table 1:** How this fits in

Advanced generalist medicine, which critically generates, uses, and appraises a whole-person understanding of illness in context, is considered a particularly suitable approach to address today’s complex healthcare challenges. However, in a primary care system that is grappling with complexity and uncertainty, GPs may not feel supported in their generalist work. Our study used an applied qualitative framework to identify grey areas within primary care systems causing tensions for GPs. We outline these tensions and grey areas, and suggest how to target them so that GPs can be better supported in their generalist work.

We know that ‘more of the same won’t fix general practice’;^20^ to keep patching the problem with the same approaches is only delaying the inevitable, and we need to start thinking and acting differently about general practice work. So, what do we need to do differently? To understand this, in the current study, we set out to answer the following question: what do GPs’ professional and educational experiences with advanced generalist medicine (AGM) tell us about changes needed to support their generalist work? Such understanding is a crucial step towards redesigning primary care services and education so that both GPs and their practices are better supported to handle the complexity of their work.

## Method

We conducted a qualitative study involving participants enrolled in Catalyst, a career development programme (CDP) on AGM for new-to-practice GPs in the Humber Coast and Vale area (UK). Catalyst is a 2-year programme (1 year at the time of the study) that addresses the theory and practice of AGM with the aim to develop expertise, using and analysing data for clinical practice, build professional networks, and engage in research-informed quality improvement work. We adopted an applied qualitative research framework, that focuses on policy and practice (generalist practice in this case), targets a specific population (newly qualified GPs enrolled in an online programme), and aims to develop actionable insights and recommendations.^21^^,^^22^

Recruitment started after receipt of ethical approval by the Hull York Medical School Ethics Committee. We recruited new-to-practice GPs (that is, within 2 years of qualifying) enrolled in the programme, as they could provide insights into AGM based on their participation in the course while facing their own distinct challenges and barriers within the healthcare system and being particularly affected by the ongoing workforce crisis.^23^ In total, we recruited 32 participants (24 women, eight men), all qualified between 2019 and 2021 (with one exception, who qualified in 2016), and whose age ranged between 31 and 49 years (averaging 35.6 years). All the participants learned about the opportunity to participate in the study when the researcher introduced herself during the first Catalyst session. As everyone on the programme was eligible to participate, we relied on volunteer sampling, hence having the enrolled GPs volunteer to participate in the interviews and/or focus groups by contacting the researcher themselves.^24^

Data collection involved 36 semi-structured individual interviews (which took between 34 and 59 min) and six focus groups (taking between 78 and 108 min). The programme had two start dates, April 2021 and September 2021, and all data collection activities were concluded by July 2022.

Of the 32 participants, 19 were interviewed at the beginning of the first year (first 3 months) and 17 at its conclusion (final 3 months), as not all of them could participate in both interviews. The focus groups were conducted halfway through the programme, with the number of participants in each group ranging from three to six (28 in total). All interviews and focus groups were conducted online and were audiorecorded for anonymised transcription. Recruitment stopped when data saturation was achieved.^25^ This meant keeping track of the topics discussed by the interviewees along with field notes (examples include ways in which they defined AGM or resources they needed to implement AGM). When no new or different perspectives were introduced by the participants, we conducted two more start-of-the year interviews and one more end-of-year interview to check for data saturation.

The interviews and focus groups guides (Supplementary Box S1 and S2) were designed to evaluate the CDP programme while addressing issues specifically relevant to the general practice workforce. Both were pilot tested, the interview guide with a foundation (year 2) doctor and the focus group guide in a meeting with the Catalyst tutors, who were all GPs.

Most of the interviews and focus groups were conducted by the first author, with a small number of interviews (four start-of-year interviews) being conducted by other members of the research and teaching team (one being the second author).

At the time of the study, the first author completed a PhD in medical sciences, was a research fellow working on the evaluation of the programme, her research experience ranging from person-centred care to medical epistemology and qualitative research. She had no prior relationship with any of the participants.

Data analysis employed a combination of predetermined and data-generated codes.^26^ Predetermined codes were based on normalisation process theory,^27^ which explains how new interventions are embedded and sustained in daily practice and informed the evaluation of the CDP. On the other hand, inductive coding was used to identify further issues of specific relevance to the participants. This mix of predetermined and data-generated coding was facilitated by framework analysis, which is particularly suited to applied health research frameworks because of its flexibility and cross-sectional focus.^28^

Following Lambert and Loiselle’s approach,^29^ the interviews’ and focus groups’ datasets were initially analysed separately by the first author (although codes and themes were refined and discussed iteratively between both authors). After the focus groups and interviews the thematic indexes were finalised; the integration process involved juxtaposing quotes from similar themes to identify further nuances or perspectives.

Evidence of tensions arose during the ‘refining themes’ stage of thematic analysis,^30^ as we were refining preliminary themes about the clinical negotiation between doctor (participant) and patient, which we developed in the context of examining the participants’ approach to generalist practice.

In framework analysis, all quotes are put in a matrix to facilitate cross-comparisons between and within participants. At this stage, we realised that one participant referred to the greyness of primary care consultations in both a favourable way (that is, because they are flexible) and an unfavourable way (because they require ‘permission’ to operate in those grey areas). We identified the contrast between these two feelings as a tension that, for theme refinement purposes, we defined as a state of conflicting thoughts, wishes, or feelings created by internal conflicts and/ or external pressures.

At this point, we engaged in the hermeneutic circle of interpretation by going back and forth between parts (codes and excerpts) and the whole (the dataset)^31^ to see if other examples of tensions and/or grey areas were present in other GPs’ professional and educational experiences.

## Results

**Figure 1.**
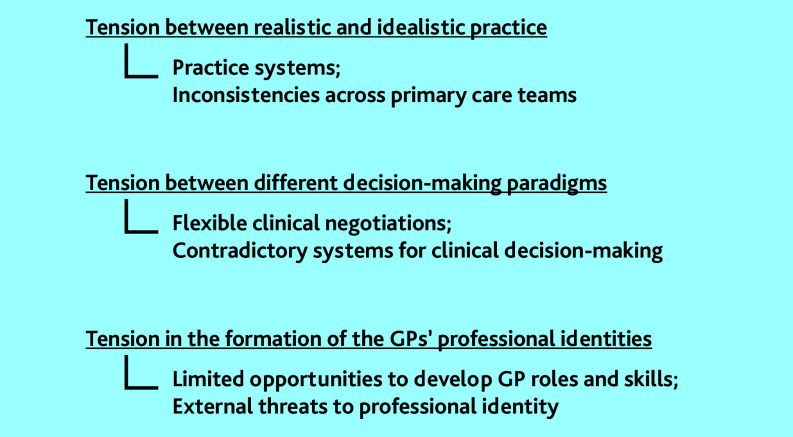
Tensions and grey areas experienced by the participating GPs in everyday practice that affected their capacity for generalist medicine.

Our analysis identified and described three themes, namely three types of tensions experienced by GPs in everyday practice that affected their capacity for generalist medicine. These tensions are listed below (Figure 1):

- tension between realistic and idealistic practice;
- tension between different decision-making paradigms; and
- tension in the formation of the GPs’ professional identities.

These tensions were hidden within grey areas of general practice, namely aspects of the participants’ experiences that were poorly defined or understood.

To preserve anonymity, pseudonyms are used for participant quotes.

### Tension between realistic and idealistic practice

This first theme manifested as a tension between the ideal application of generalist medicine in practice and the challenges that the participants experienced as they tried to achieve that:

*‘I think* [AGM is] *in a grey zone between what we can actually, realistically achieve, and what’s kind of idealistic.* […] *No one would disagree that* […] *it’s best to delve into the psychosocial aspect of someone’s life,* […] *but can you realistically do that in the middle of crazy clinics where you’ve got 10 minutes or 5 minutes?’*

(Ben, initial interview)

Ben describes a grey area within the clinical consultation where the adoption of generalist approaches is perceived to be only partial. The tension becomes apparent when he recognises that the systems within the practice (for example, ‘crazy clinics’) relegate AGM to an idealistic state, whereas it should be the preferred approach instead (‘no one would disagree that [...] it’s best’).

These feelings were echoed by Lisa, who felt the system in which she worked did not support generalist practice:

*‘Of course the ideal is to do this* [practice generalist medicine]*, and we know that this is good medicine, but when you’re in a broken system, how far do you go in your own time to try and make things at the standard they should be?’*

(Lisa, initial interview)

Just like Ben, Lisa refers to a generally acknowledged (‘we know’) ideal form of medicine, but also feels that it is the broken system itself that prevents the achievement of a high standard of care, rather than her own choice.

However, another GP highlighted how it is not only the system that can hinder generalist approaches, but also inconsistencies across the practice’s staff:

*Everyone* […] *needs to be thinking in the same sort of way, or thinking about the same sort of concept;* […] *so* [AGM is] *more of an ideal thing that doesn’t necessarily translate fully into practice, because everyone’s thinking about different things, or pulled in different directions; the basis of it is there in the real world, but it’s never quite fully formed.’*

(Alex, final interview)

This shows how AGM can feel confined to an ‘ideal’ approach when colleagues at the practice do not prioritise such a type of care. In this example, the lack of a clear, shared strategy or vision across the healthcare team emphasises the perception that AGM cannot be realised only through individual efforts but needs to become part of the broader organisational culture.

### Tension between different decision-making paradigms

Another tension related to conflicting decision-making paradigms. In these instances, the participants felt compelled to rely on decision-making paradigms that they believed to be inadequate to address the health problems of their patients. The potential for this tension lay in what Jane called the ‘glorious grey bit’ of general practice, namely that flexibility required for the achievement of whole-person care:

*‘In medicine and surgery, there’s usually a very clear right thing to do* […]*, and in general practice we have this kind of glorious grey bit where it’s not black and white.* […] *There is a bit where you negotiate with your patient, you explain to them, “this is what we should do, this is what you have said you would like, this is what we could do in the middle”.’*

(Jane, final interview)

The grey area described by Jane presents an opportunity for patients and doctors to collaborate, leveraging each other’s insights to establish a shared understanding. This grey zone sets generalist practice apart from specialist approaches, yet it also exposes tensions between divergent decision-making paradigms, as highlighted by other interviewees. In particular, there was a tension between reliance on single-disease clinical guidelines, which the participants perceived to be the system’s preferred approach and the need for more adaptable approaches that accommodate for complexity. According to some participants, the origins of this tension could be traced back to medical education:

*‘They tend to give you cases which are useful for your exams, or which are easy to deal with, like sore throat, or asthma, or diabetes* […]*. But once I’ve started to practise as an independent GP, it’s different, I don’t see the patients come with just one condition.’*

(Zara, initial interview)

Medical education as experienced by this GP emphasised the knowledge and implementation of guidelines. Upon entering practice, this participant experienced a dissonance between the guidelines-oriented system they trained within, and the complex conditions presented by her patients.

The system’s nudge towards certain decision-making paradigms was felt as an imposition when adopting different approaches could come at a cost:

*‘You try to follow all the guidelines, all these things in order to avoid any problem, any mistake, any problem with the GMC* [General Medical Council]*, and you forgot that you are doctors, and you have a lot of knowledge, and you could go further than these guidelines.’*

(Ana, focus group)

Ana highlighted that reliance on guidelines could stem from a sense of obligation rather than a genuine belief that it is in the best interest of the patient, which caused some of the GPs in this study to feel disconnected from their own expertise and clinical judgement.

### Tension in the formation of the GPs’ professional identities

This final theme relates to how the GPs defined their professional identities. These were threatened by a perceived negative perception of GPs, which positioned them as inferior to other specialties:

*‘I think we’ve had years of the “just a GP” comment being thrown at us and, and actually using it* [ourselves] *as well. My husband’s a doctor, but he saves life and limb and I’ll –* [laughs] *– I’ll hear myself go “Oh, I’m just a GP”. Why am I just a GP?* [laughs] *Why, why, why do I say that? But actually, it has traditionally been very hard to speak about the hard work of general practice.’*

(Nadia, initial interview)

Nadia reflected on the evolving perception of her own profession, which underwent a transformation triggered by external comments about being ‘just a GP’. With time, she internalised this sentiment, and started using that same comment to describe herself. However, threats to the GPs’ professional identity were multiple, as also explained by Nia and Esther:

*‘It’s easy to forget what general practice actually means.* […] *Sometimes, in the pressure of things, we forget what our jobs actually are, and we just tend to troubleshoot and get on with the day and be ready for another day; so it’s like a battle that we’re continuously fighting’.*

(Esther, final interview)

*‘Due to some factors that are beyond our control,* […] *like* […] *every appointment has to be 10 minutes, or the troubles we have with the media where patients don’t feel we know enough to help them,* […] *our secondary care colleagues who also kind of promote that idea that a GP is not an expert* [in] *anything* [laughs]*, they just see us as, you know, you’re just someone who knows a little bit of things and you’re not an expert. Through the years,* […] *it tends to start rubbing off on the GPs themselves, and they start looking down on their skills and seeing themself as “well, I’m not an expert, I’m just a GP”.’*

(Nia, final interview)

As both Nadia and Nia pointed out, these external pressures gradually seeped into their own professional identities. As Nia talks about 10-min appointments, Esther explains how the ‘pressure of things’ limits GPs to only do some troubleshooting, hence constraining their opportunities to develop their GP roles and skills beyond that. The recurring phrase ‘just a GP’ and related self-deprecating remarks on GPs’ professional roles were used by several (*n* = 7) participants, further reinforcing this disempowered self-perception.

Throughout these examples, the tension lies in the GPs’ struggle to maintain a clearly defined and empowering sense of professional identity. This struggle is juxtaposed against the pressures, initially external but eventually internalised, that eroded their confidence and undermined their professional self-image.

## Discussion

### Summary

This study set out to explore GPs’ experiences with AGM to understand how to support their generalist work. Thematic analysis revealed three tensions experienced by the participating GPs: tension between realistic and idealistic practice; between different decision-making paradigms; and in the formation of the GPs’ professional identities (Figure 1).

For example, the tension between idealistic and realistic practice related to practice systems that did not support what the GPs in this study thought was ideal generalist care and the lack of shared strategies across primary care teams. The tension between different decision-making paradigms emerged as the flexibility of general practice consultations made contradictory systems for decision making apparent (for example, biomedical versus biopsychosocial models of care). Finally, tensions in the formation of professional identities were related to external threats to professional self-image (such as the perception of the mass media depiction of general practice services) and to the participants’ limited opportunities to develop their GP roles in practice while feeling devalued in comparison with their specialist colleagues.

### Strengths and limitations

This study has some limitations. For example, it involved only newly qualified GPs, hence missing the perspectives of GPs at different career stages, or other healthcare professionals, who might have their own challenges and experience different tensions. Furthermore, these findings were developed in the context of the evaluation of a career development programme. Therefore, further research that explicitly aims to involve GPs at other career stages or that seeks to identify how tensions are generated or unfold in practice (for example through observations) may help unveil and address more grey areas in general practice.

On the other hand, a strength of this study resides in the richness of data achieved through conducting both focus groups and individual interviews. Moreover, this study benefitted from the unique insights contributed by GPs who possessed an understanding of AGM, by virtue of their involvement in the CDP from which they were recruited. Consequently, we were able to offer a distinctive perspective on the impact of these approaches and on their potential to shape healthcare services when adopted more widely.

### Comparison with existing literature

The grey areas described in this study were characterised by a lack of clarity surrounding the concepts, rules, and roles of general practice and of the knowledge work of AGM. With knowledge work, we mean how knowledge (from research evidence to patient experience) is discovered, incorporated, and generated into daily practice in the management of the patient’s conditions.^32^ However, this study showed how the knowledge work of AGM is insufficiently recognised in the current primary care system, as also reflected by some services’ configurations leading to limited patient–doctor continuity, reliance on quality parameters that prioritise the use of single-disease guidelines,^33^^,^^34^ or short (for example, 10 min) consultations in which patients and doctors can only discuss one medical concern, hence limiting doctors’ capacity to address complex problems.^35^

These infrastructures, however, collide with primary care’s own call for more comprehensive, whole-person approaches.^36^^,^^37^ Whereas it has been argued that system innovations in general practice should aim to acknowledge person-centred perspectives and integrate them with biomedical knowledge,^38^ the participants in this study experienced a clash between the two instead. The tensions they experienced as a result can be explained through Henriques’^39^ account of the failure to reconcile coexisting ‘mini-epistemologies’ (that is, different ways to determine which knowledge is valid), which hinders the accumulation of understanding necessary for managing complex healthcare challenges.

Compounding the issue of the limited recognition of generalist knowledge work is the already denounced marginalisation of general practice compared with other specialties, and the discrediting of GPs’ knowledge in their interactions with other medical professionals.^40^ In this context, recognising the skills and knowledge of GPs is crucial to advance primary care, as systemic change relies on bottom-up initiatives that target areas of tension within established systems.^41^ As this study identified such tensions within some inconsistencies that are intrinsic to primary care, these initiatives need to focus on transforming knowledge and perspectives.^42^ Fostering active participation of GPs in medical education and research, and embracing a plurality of perspectives in the design of healthcare services can help work towards this aim.^43^ Still, medical education requires reform as Wenzel^44^ argued that we need to change the focus of medical education from what we know to how we use what we know, for example by fostering the students’ curiosity, inquiry, and reasoned doubt, and praising their questions rather than their answers. Whereas this study’s findings reiterate that some participants felt they were trained to pass their exams, rather than be prepared for real-life clinical practice, work conducted in the field of primary care education emphasises how current approaches to assessment still do not fully prepare medical students for the inevitable uncertainties of clinical practice.^45^

### Implications for research and practice

Our findings identify areas of change needed in training and service design to tackle the tensions and so support new-to-practice GPs in their generalist work. Specifically, we identify two areas of change needed:

- legitimising the knowledge work of AGM; and
- defining clear and empowering professional identities throughout both education and practice.

First, to legitimise the knowledge work of AGM, it is paramount to make its expertise visible to primary care stakeholders, from policymakers to medical students, the mass media, and health professionals themselves. We know from previous research that opportunities for successful change can arise when health providers are enabled to actively engage in healthcare initiatives.^46^ This could mean redesigning primary care services to prioritise care work,^47^ or involving newly qualified GPs in the design of their own education and research to shape healthcare’s decision-making paradigms. In medical education settings, newly qualified GPs can build on each other’s knowledge by sharing their clinical experience and developing contextual knowledge needed to address the complexities of real-life clinical decision making.^48^ On the other hand, promoting academic primary care and enhancing the scholarship and research skills involved in generalist practice will promote clinical conceptualisation, research in practice settings, and retention.^49^

Finally, promoting a positive professional identity begins by challenging (self-) deprecating language that threatens newly qualified GPs’ self-esteem and by dispelling false equivalences such as that of the GP as a ‘Jack of all trades and master of none’.^50^ Several ways to challenge denigratory language have already been suggested, from enhancing understanding of both primary and secondary care roles, to bringing attention to the consequences of denigratory comments.^51^ Further efforts can also be made to recognise and make visible the breadth of knowledge and skills GPs possess. Establishing communities of practice and varied healthcare networks focusing on AGM can help GPs get together and develop leadership and advocacy skills,^52^^,^^53^ while also affirming their own role and expertise.
